# The Relationship Between Spinal Cord Atrophy and Cardiovascular Risk

**DOI:** 10.7759/cureus.88072

**Published:** 2025-07-16

**Authors:** Tejen Shah, Louis Salciccioli, Seyed Zaidi, Kamal Akbar, Shabnam Seydafkan, Lily Lee, Elizabeth Helzner, Gulzhan Tologonova, Srinivas Kolla, Jason Lazar

**Affiliations:** 1 Cardiology, SUNY Downstate Health Sciences University, Brooklyn, USA; 2 Pathology, SUNY Downstate Health Sciences University, Brooklyn, USA; 3 Epidemiology and Biostatistics, SUNY Downstate Health Sciences University, Brooklyn, USA; 4 Radiology, SUNY Downstate Health Sciences University, Brooklyn, USA

**Keywords:** atherosclerotic cardiovascular disease risk score, cardiovascular risk factors, diabetes, hypertension, spinal cord atrophy

## Abstract

Objectives: Lower brain volume is associated with various cardiovascular (CV) risk factors, but less is known about the spinal cord (SC). Concomitant SC atrophy may contribute to motor weakness and slowness that are prominent features of frailty, known to increase mortality. The objectives of this study were to determine potential relations between SC size, age, individual CV risk factors, and overall CV risk.

Methods and results: We retrospectively reviewed 121 patients (age 60 ± 13 years, 75.2% females) who were referred to our institution for magnetic resonance imaging (MRI) of the cervical SC. Patients with known degenerative neurological or congenital disease were excluded from review. CV risk factors were obtained from medical records, and the Atherosclerotic Cardiovascular Disease (ASCVD) risk score was calculated. Cross-sectional SC area (SCA) was traced on each slice of the C2-C6 cervical images with electronic calipers and averaged. Mean SCA was inversely correlated with age (r = -.19; p = .04) and creatinine level (r = -.20; p = .03), but not with height (r = .04; p = .69), weight (r = .03; p = .72), or body mass index (r = .02; p = .80). There was a stepwise decrease in SCA in patients without hypertension (HTN) or diabetes mellitus (DM) (n = 23) compared to those with only HTN (n = 55) (84.4 ± 9.4 mm^2 ^vs 79.6 ± 11.2 mm^2^) and to patients with DM and/or HTN (n = 43) (84.4 ± 9.4mm^2^ vs 76.6 ± 8.3mm^2^ (p = .01). On multivariate regression, DM was an independent predictor of lower SCA (β = -4.4; p = 0.03), and there was a trend toward lower SCA in patients with only HTN (β = -4.0; p = 0.1) (p = 0.02 for the multivariate model after adjusting for age and creatinine). Among 74 patients with ASCVD risk scores, SCA had a moderate inverse correlation with the ASCVD score (r = -.42; p < 0.001), which remained an independent predictor of lower SCA on multivariate analysis (β = -2.9; p = 0.002).

Conclusion: Lower SCA appears related to existing DM and possibly HTN, as well as overall CV risk. SC atrophy in patients with CV disease and risk factors may contribute to frailty, which is associated with increased mortality. This study is subject to the limitations of a retrospective cross-sectional study of a relatively small sample size.

## Introduction

Lower brain volume has been found to be associated with various cardiovascular (CV) risk factors and higher overall CV risk, and in turn, brain atrophy has been linked to cognitive dysfunction [1,2]. Less is known about atrophy related to CV risk factors of the other component of the central nervous system (CNS), the spinal cord (SC) [3,4]. Brain and SC size appear to be interrelated, as a postmortem study found SC size highly correlated with total brain volume, and another study found total brain volume to be the major determinant of upper cervical SC area in living humans [5,6]. While allometric scaling and genetic factors have both been proposed to explain the close relation between brain and SC size, whether CV risk factors individually affect the brain and SC has been largely ignored [6-8]. Most prior studies evaluating SC size have been conducted using magnetic resonance imaging (MRI) in patients with multiple sclerosis, a neurodegenerative disorder [9,10]. Several studies have also assessed SC size in the setting of diabetes, which is well-known to induce peripheral neuropathy but is also a robust CV risk factor [11-13]. Accordingly, the objectives of this study were to determine potential relations between SC size and (1) age, (2) individual CV risk factors, and (3) overall CV risk. Specifically, we hypothesized that aging and CV risk factors would be associated with lower mean SC cross-sectional area (SCA) derived from MRI. Although there may be neurological effects on the SC size, this study focused on the effects of CV risk factors. The functional significance of SC size in relation to CV risk and overall CNS health may be important, since increased CV risk results in SC atrophy would negatively impact overall CNS health and possibly increase frailty and therefore mortality. 

## Materials and methods

This study was approved by the Institutional Review Board of the State University of New York Downstate Medical Center. A convenience sample of consecutive patients aged >30 years who were referred to the University Hospital of Brooklyn for MRI studies of the cervical spine over a 13-month period was considered for enrollment into this retrospective study. This study is subject to the limitations of a single-center study, and as most patients were African-American, the results may not be generalizable to other populations. Patients were excluded from our analysis if they were completely lacking clinical information; had paralysis or suspected or confirmed neuromuscular disorders, scoliosis, or a diagnosis of congenital spinal abnormalities such as syrinx or Chiari Malformation; had known malignancy; or were pregnant [14]. Clinical data, including past medical history and social history, were obtained from the electronic medical record. Patient data included age, sex, race, any previous smoking history, cholesterol (total, high density lipoprotein (HDL), low density lipoprotein (LDL) and triglycerides (TG)), most recent blood pressure (BP) to the MRI study, history of diabetes mellitus (DM), history of hypertension (HTN), history of hyperlipidemia, height, weight, body mass index (BMI), prior stroke, known coronary artery disease (CAD) defined as previous documented myocardial infarction (MI), abnormal stress test results or >50% stenosis by coronary angiography, history of congestive heart failure (CHF), and other chronic disease states. DM, HTN and hyperlipidemia were determined by documentation of the established diagnosis on electronic medical record, current treatment with medication (cholesterol lowering medication taken in the absence of CAD), or by biomarker evidence (fasting glucose levels of >126 mg/dL, BP of ≥140/90 mmHg, plasma cholesterol level of ≥240 mg/dL). The specific indication for the MRI was also recorded (neck pain with or without radiculopathy or other neurological symptoms/myelopathy, posttrauma, preoperative evaluation). Indications were then grouped into the following three categories: (1) pain without neurological symptoms, (2) neurological symptoms with or without pain, and (3) trauma.

Global CV risk was assessed using the American College of Cardiology atherosclerotic CV disease risk score calculation (age, sex, race, systolic and diastolic BP, cholesterol (total, LDL, HDL)), which predicts the 10-year risk of first atherosclerotic CV event [15]. Using this risk calculation, people are classified based on estimated risk for a CV event, with the 10-year risk <5% considered low risk, 5%-7.5% borderline risk, 7.5%-20% intermediate risk, and ≥20% high risk. For study purposes, we used the calculated risk score as a linear variable.

**Figure 1:**
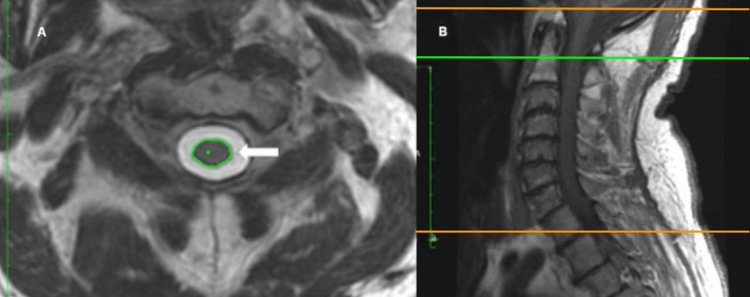
Spine MRI axial T2-weighted image at the level of mid-C2 vertebral body shown on the left. Automatic segmentation tool (arrow) was used to acquire cross-sectional areas of the spinal cord at mid-C2 to C7 vertebral levels (panel A). Midline sagittal T1-weighted image is shown on the right with reference lines (panel B) MRI: magnetic resonance imaging

All patients underwent SC imaging on a GE Optima 450w 1.5 Tesla MR scanner equipped with GEM Suite Head Neck Spine array (GE Healthcare, United States). The protocol adhered to standardized guidelines or best practices for SC imaging. Cervical SCA was measured from T2-weighted MRI images according to published recommendations [16]. Measurements were made by a single experienced physician who was blinded to the clinical data. The outline of the SC was traced at the middle of each vertebral body from C2-C6 using OsiriX DICOM viewer software, using the range of interest (ROI) function. This function automates SC tracing in a manner similar to electronic calipers with area readings directly displayed (Figure 1).

The SC outline was visually inspected to ensure accuracy. If an individual SC level was deemed unclear and unable to be accurately measured, it was excluded, and patients with >3 SC levels unable to be measured were excluded from the analysis. The traced SCAs from each of the MRI slices were then averaged. Repeated MRI measurements of SCA were determined on two separate occasions by the same observer and by a second experienced observer (trained by a neuroradiologist) to assess intra-rater and inter-rater reliability.

Continuous variables are expressed as mean ± standard deviation. Categorical variables are presented as percentages. The Kolmogorov-Smirnov test was used to test the mean SCA for normality. The independent t-test was used to assess differences in mean SCA between patients with and without each of the CV risk factors. Analysis of variance (ANOVA) was used to compare the mean SCA in patients without risk factors to those with HTN and those with DM and HTN. The Spearman correlation was used to assess potential relations between continuous variables. Significant variables in the univariate analysis were included in the multivariate analyses to determine independent predictors of mean SCA. Covariates included age, sex, race, BMI, history of HTN, DM, prior to current smoking history, and the clinical indication for the MRI study. All statistical analyses were performed using IBM SPSS Statistics for Windows, Version 21 (Released 2012; IBM Corp., Armonk, New York, United States). Two-tailed p-values of <0.05 were considered statistically significant. Intra-observer and inter-observer reliability for the SCA were determined by the intraclass correlation coefficients (ICC) and were found to be 0.98 and 0.99, respectively.

## Results

After excluding two patients who had studies deemed uninterpretable, the study group consisted of 121 participants. The mean age was 60.5 ± 13 years, 75.2% of subjects were females, and 95% were African-Americans. Patient characteristics are shown in Table 1.

**Table 1:** Study participant characteristics (total N = 121) ASCVD: Atherosclerotic Cardiovascular Disease

	Male	Female	All patients
African-American	28 (24.3%)	87 (75.7%)	115 (95%)
White	1 (33.3%)	2 (66.7 %)	3 (2.5%)
Asian	1 (50%)	1 (50%)	2 (1.7%)
Other	0	1 (100%)	1 (0.8%)
Age (years)	30 (60.2 ± 11.3)	91 (61.2 ± 13.4)	60.5 ± 13
Body mass index (BMI) (kg/m2)	29 (27.9 ± 6.0)	88 (31 ± 8.0)	30.5 ± 7.7
Systolic blood pressure (mmHg)	30 (130 ±17)	91(132 ± 16)	130 ± 16
Diastolic blood pressure (mmHg)	30 (74 ± 11)	91 (74 ± 10)	74 ± 11
Creatinine (mg/dl)	29 (1.7 ± 2.2)	90 (1.2 ± 2.0)	1.3 ± 2.0
Total cholesterol (mg/dl)	18 (153 ± 44)	64 (184 ± 42)	177 ± 44
Diabetes	9 (30%)	34 (37%)	35.5%
Hypertension	21 (70%)	74 (81%)	78.5%
Hyperlipidemia	8 (26.7%)	50 (54.9%)	47.9%
Smoking history	11 (36.7%)	16 (17.6%)	22.3%
Prior myocardial infarction	2 (6.7%)	5 (5.5%)	5.8%
Coronary artery disease	5 (16.7%)	10 (11%)	12.4%
Prior stroke	4 (13.3%)	12 (13.2%)	13.2%
Congestive heart failure	2 (6.7%)	5 (5.5%)	5.8%
Mean ASCVD risk score (n = 74)	16 (20.9 ± 12.4)	58 (15.5 ± 12.2)	16.6 ± 12.3
Mean spinal cord area (mm)	30 (79.9 ± 12.4)	91 (79.3 ± 9.4)	79.5 ± 10.2

For the entire cohort, the mean SCA was normally distributed and measured (79.5 ± 10.2 mm^2^). For the entire cohort, the mean SCA was inversely correlated with age (r = -.19; p = .04) and creatinine level (r = -.20; p = .03), but not with height (r = .04; p = .69), weight (r = .03; p = .72), or BMI (r = .02; p = .80) (Table 2). 

**Table 2:** Spearman correlation coefficients of continuous variables and SCA ASCVD: Atherosclerotic Cardiovascular Disease; SCA: spinal cord area; * indicates p < 0.05

Variable	r value	p-value
Age	-0.19*	0.04*
Body mass index (BMI)	0.02	0.80
Creatinine	-0.20*	0.03*
Hemoglobin (Hb)	.05	.61
Total cholesterol	0.004	0.97
Low density lipoprotein (LDL)	0.03	0.81
High density lipoprotein (HDL)	-0.13	0.15
Triglycerides	-.11	.32
ASCVD risk score	-0.42*	<0.001*

**Figure 2:**
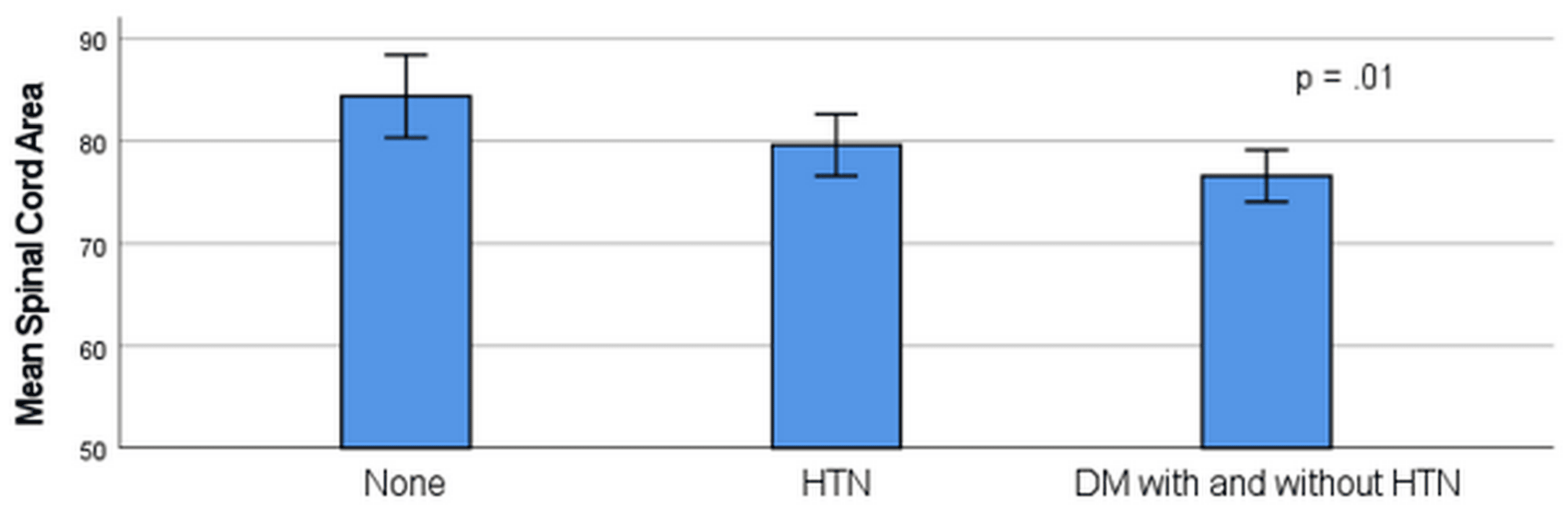
Relationship of the spinal cord area and cardiovascular risk factors (N = 121) HTN: hypertension; DM: diabetes mellitus; p-value determined using the one-way analysis of variance (ANOVA) test

There were no significant differences in SCA for those with a smoking history, hyperlipidemia, history of CAD, or MRI indication. There was a stepwise decrement in the mean SCA from those without either HTN or DM (n = 23) to patients with only HTN (n = 55), and to those with DM plus HTN or only DM (n = 43) (84.4 ± 9.4 mm^2^ vs.79.6 ± 11.2mm^2^ vs. 76.6 ± 8.3mm^2;^ p = .01) (Figure 2). 

On multivariate regression including significant univariate predictors of age and creatinine, the independent predictor of the mean SCA was DM (β= -4.4; p = 0.03), and there was a trend toward HTN (β= -4.0; p = 0.11) (p = 0.02 for the model; adjusted R^2^ = 0.06) (Table 3).

**Table 3:** Univariate and multivariate analyses of SCA and predictive variables SCA: spinal cord area; * indicates p < .05

Variable	Univariate analysis		Multivariate analysis	
	r value	p-value	β value	p-value
Age	-0.19*	0.04*	-0.025	0.75
Creatinine	-0.20*	0.03*	-0.027	0.95
Hypertension only	0.12	0.20	-0.40	0.11
Diabetes and hypertension	-0.30	<0.001*	-0.44	0.03*

**Figure 3:**
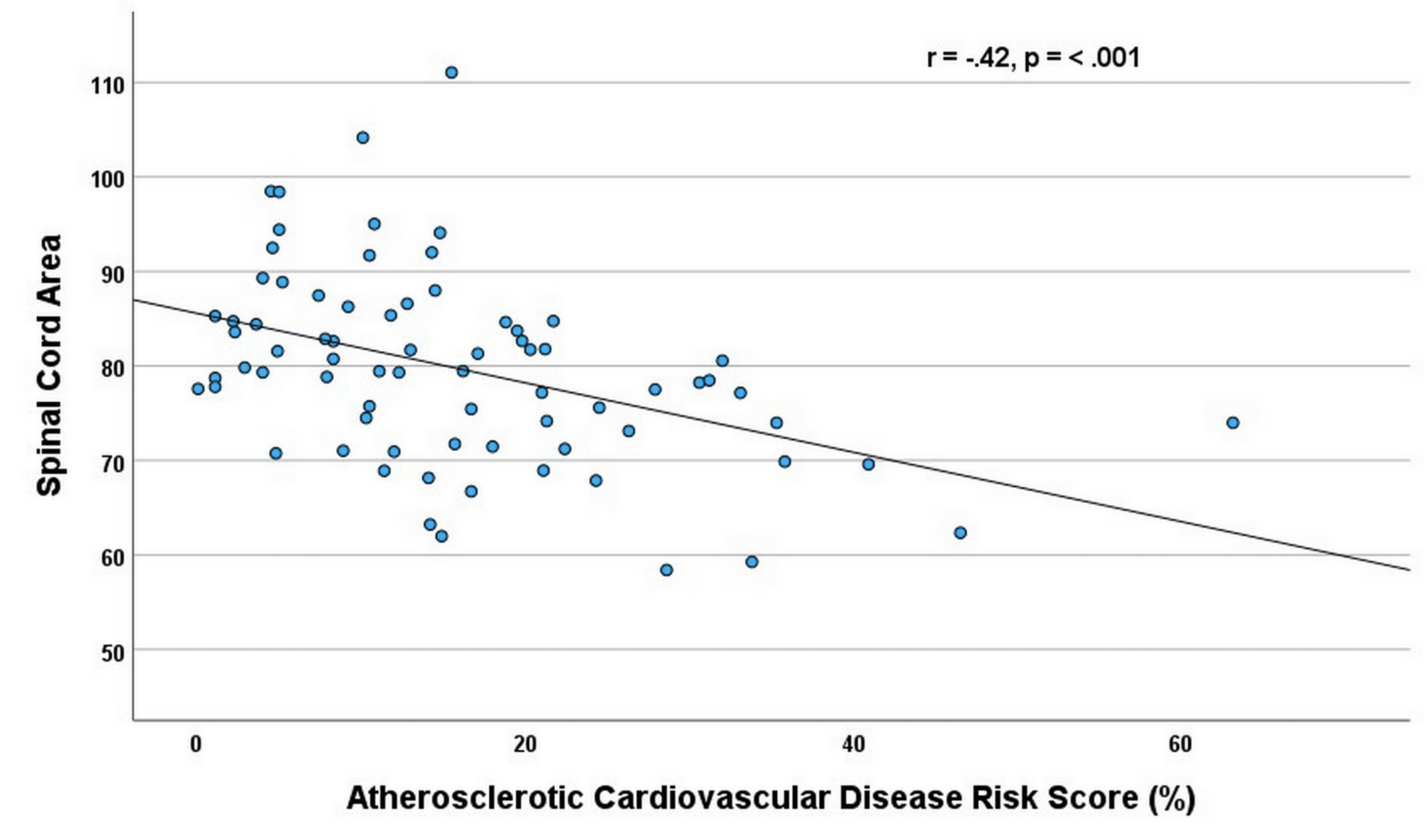
Relationship of the spinal cord area (mm) and atherosclerotic risk score (n = 74) p-value was determined by Spearman correlation

The addition of MRI indication categories and BMI to the model did not significantly change the results. Among the 74 patients with complete data to calculate the Atherosclerotic Cardiovascular Disease (ASCVD) risk score, there was a moderate inverse correlation with SCA (r = -.42; p < 0.001) (Figure 3).

On multivariate analysis adjusting for creatinine level (β= -.29; p = 0.003) (p = .007 for model) or adjusting for both creatinine and MRI indications (β = -.27; p = 0.004) (p = .004 for model), higher ASCVD remained an independent predictor of lower SCA.

## Discussion

The major finding of the present study was that both DM and a higher ASCVD score were independently associated with lower mean SCA in community patients referred for cervical MRI studies. Patients with HTN also had a lower SCA than those without, although HTN only reached a trend toward significance on multivariate analyses. The decrease in SCA for these variables is illustrated in Figures 2, 3. These findings were independent of age, creatinine, and BMI. Notably, SCA was inversely related to the ASCVD score, a commonly used, simple, and inexpensive method of CV risk assessment. The stepwise decrement in SCA is possibly due to the increasing effect of CV risk factors on the SCA. This suggests that an increasing burden of CV risk factors would have detrimental effects on the SCA. There were only three patients with DM and not HTN, and so analysis of DM alone was not possible. This is also consistent with the strong inverse correlation of the SCA with the ASCVD score, which assesses overall risk.

Although measurement of SCA has long been used to assess SC atrophy in the setting of multiple sclerosis [8-10], data relating SC size to CV risk factors and overall risk are sparse. We are unaware of studies assessing specific CV risk factors other than those addressing diabetes, a well-known cause of neuropathy [11-13]. The majority of studies have found smaller SC size in diabetic patients with, but not without, polyneuropathy [12,13]. Accordingly, the mechanism of SC atrophy in the setting of diabetes has largely been assumed to be neuropathic. However, several lines of evidence would support a vascular etiology. Firstly, we assessed and found an association between higher global CV risk and lower SC area. Also, several prior studies have demonstrated the presence of atherosclerosis within the spinal arteries, and other studies have documented pathological findings indicative of ischemic myelopathy [17-22]. Furthermore, prior studies have directly attributed progressive myelopathy, lower back pain, lumbar disc degeneration, and vertebral body infarction to chronic SC ischemia [23-25]. Given these findings, it appears to date as though the potential for chronic ischemic SC syndromes has been underappreciated, possibly because the spinal arteries are widely understood to be densely collateralized [3,4,17-19]. Of note, chronic cardiac ischemic syndromes are not uncommon despite the heart similarly possessing a dense system of collateral vessels [26]. Another interesting finding to support a vascular etiology is that lower SCA was associated with HTN on univariate analysis, and we found a stepwise decrease in patients without either HTN or DM, to HTN, and finally to those with DM. While HTN only reached a trend toward significance on multivariate analysis, the vast majority of patients had HTN (79%), leaving a small comparison group of subjects without HTN, and all but three patients with DM also had HTN. While diabetes is widely viewed to portend microvascular complications [13], HTN, which is strongly associated with cerebral atrophy, is also known to result in clinical manifestations related to microvascular disease [26]. That we found only a trend between SCA and HTN may be due to our small sample size, and this warrants further study. Additionally, the study of other biological markers of vascular disease and risk factors may add further insight into the mechanisms of lower SCA and the clinical implications. In addition, longitudinal studies may address disease progression and add to the knowledge of the clinical significance of lower SCA.

The involvement of microvascular disease could potentially explain the significant relation between mean SCA and CV risk, but not known CAD in this study. A potential role of microvascular disease in the pathogenesis of SC atrophy is further suggested by an autopsy study of older subjects with and without Parkinson’s disease that found more extensive microvascular alterations in the SC than in the brain [21]. Of note, SC white matter changes associated with the severity of spinal arteriosclerosis were found to be associated with spinal white matter pallor [21]. In another study, microvascular disease at the SC level was found to contribute to diabetic neuropathic pain [13]. Moreover, the microvasculature of the SC has been well-described and implicated as a culprit of SC pathology in a prior study [27]. In addition, HTN may alter cerebrospinal fluid homeostasis, as evidenced by increased arterial pulsations resulting in increased spinal fluid tracer movement in the SC parenchyma with potential downstream effects on the SC itself [28]. Nonetheless, the interplay between HTN, microvascular disease, and SC atrophy merits further study.

Several other study caveats merit further comment. The mean SCA was measured at the cervical level, consistent with most prior studies, and the mean values measured in this study are similar to those previously reported. The lack of association of the mean SCA with height, weight, and BMI is consistent with prior studies [5]. While the mean SCA was associated with global CV risk as measured by the ASCVD risk score, it was related to some but not all CV risk factors. Specifically, SC size was unrelated to hyperlipidemia and smoking, which may be due to the small number of patients in our study with these risk factors. Variable cumulative burdens as well as treatment with lipid-lowering drugs might have obscured such relations, and future studies may characterize risk factor burdens more stringently. Of note, the ASCVD includes BP and lipid values rather than binary distinctions of CV risk factors. Also, while we did not assess peripheral neuropathy, the present study results are consistent with and extend the observations of prior studies focusing on DM in several ways. Firstly, we considered other CV risk factors and found a trend toward lower SCA in patients with HTN. In addition, as aforementioned, we evaluated the overall CV risk. Also, as compared to the prior studies, our sample size was relatively large. Lastly, the vast majority of subjects were African-Americans, an understudied group of patients with regard to SC size. Since our study involved African-Americans, a similar investigation in other populations is therefore warranted. We found creatinine significant on univariate analysis but not on multivariate analysis. Renal dysfunction is a known risk factor for vascular disease, and creatinine may be a less accurate measure of this. A larger sample size or using a more accurate measure of renal dysfunction would be of interest and may warrant further investigation. 

The clinical significance of smaller SC size remains to be determined. Given that cognitive decline has been linked to both brain atrophy and to frailty, it is intriguing to postulate that concomitant SC atrophy might contribute to motor weakness and slowness that are prominent features of frailty, a condition of growing concern that is present in up to 60% of CV patients and predictive of higher mortality despite its underlying etiology being poorly understood [29]. Nonetheless, the frequent co-existence of frailty and cognitive dysfunction suggests that underlying brain and SC pathology might be related. In support of this view, a study of subjects with multiple sclerosis found medulla oblongata volume highly correlated with upper cervical SC volume and concluded that medulla oblongata volume is associated with disability in MS and can serve as a biomarker of SC damage [30].

This study was subject to the limitations of a cross-sectional study. CV risk factors were largely determined from the electronic medical record. There was insufficient data to evaluate glycemic control. These results apply mainly to the African-American population. We related lower mean SCA to SC atrophy, but it is unknown whether there is predominantly demyelination or neuronal loss, given that both findings have been demonstrated previously [26]. In this regard, we measured the entire cord and not the white and gray matter components, which merits further study. Contemporaneous serum lipid values and the ASCVD risk score were not available in all subjects.

## Conclusions

We conclude that lower mean cervical SCA is associated with DM and possibly HTN among community patients referred for cervical MRI studies. There was a trend toward patients with HTN having lower SCA than those without. Moreover, SCA was inversely related to global CV risk measured by the ASCVD score, which is a commonly used method to calculate CV risk in clinical practice and research. These findings were independent of age, BMI, and MRI indication. That we found only a trend between SCA and HTN may be due to our small sample size, and so, this warrants further study. SC atrophy, like brain atrophy, may increase frailty and thus mortality. Studying other biological markers of vascular disease and evaluation of microvascular disease may add further insight into the mechanisms of SC atrophy and the clinical implications. In addition, longitudinal studies may address disease progression, the association of increasing SC atrophy with frailty, and perhaps add knowledge of possible treatment modalities. To our knowledge, this is the first study demonstrating higher CV risk associated with SC atrophy. Additional studies are required to confirm these findings as well as to determine the clinical ramifications, specifically whether a lower SC area is related to frailty.
